# Supplementary material for: Awareness of treatment: A source of bias in subjective grading of ocular complications
Source: PLoS One. 2019 Dec 26;14(12):e0226960. doi: 10.1371/journal.pone.0226960 (PMC6932789; doi:10.1371/journal.pone.0226960)
Supplement: Attachment — Submitted filename: Response_to_Reviewers.docx [file pone.0226960.s001.docx]

**Answers to the Reviewers’ comments and list of changes to the manuscript**

Manuscript No.: **PONE-D-19-17494**

Title: Awareness of disease progression: a source of bias in subjective grading of ocular complications

PLOS ONE

All changes are marked in red in the manuscript. We would like to use this opportunity to express our gratitude to the reviewers for their comments and suggestions, with which we believe our revised manuscript has been improved.

**Academic Editor**

Please address all reviewers’ comments below, including the following issues:

1. The background of the study is not clearly described in the Introduction. How does 'bias' differ from 'variability in grading'? Bias needs to be clearly defined and its sources detailed in the Introduction. There is a recent article on philosophical bias which the authors could also refer to in their deliberations on this area (Andersen et al. eLife 2019;8:e44929. DOI: https://doi.org/10.7554/eLife.44929). This article also notes the Catalogue of Biases (https://catalogofbias.org/about/) which is useful in the context of the current study.

ANSWER: Thank you for this comment and for providing us with very relevant sources of information to better address the subject of bias. We have edited our introduction extensively to describe with more detail and thoroughness the background of the study.

2. The concept of bias is not novel, but rather more appreciated within the clinical and scientific community (see point 1. above).

ANSWER: Indeed, we have attempted to provide a better description of relevant sources of bias. In addition, we have removed the term “novel” from the manuscript.

3. Please revise the title. The participants are not given details on disease progression, but rather images with descriptors of 'treated' and 'not treated'. The study is thus addressing the impact of awareness of treatment.

ANSWER: We have changed the title to: “Awareness of treatment: a source of bias in subjective grading of ocular complications”.

4. Please confirm that consent from the participants was given, and also provide the IRB number in the text.

ANSWER: Consent was obtained from all participants of the study. The IRB details are provided in the text.

5. Please see Reviewer 2 comments regarding the statistics used in this study. These comments must be addressed.

ANSWER: We have revised our statistical analysis and removed those parts that were redundant in accordance to the suggestions of Reviewer 2.

6. All participants were students within an Optometry program, and as noted in the Discussion, this limits the real-world relevance of the study. This issue should be emphasised in the Discussion.

ANSWER: Although this limitation of our study was already stated in the original manuscript, we have stressed the relevance of this issue.

7. There are sections in the Discussion that do not directly relate to the current study (see Reviewer 2). Please review these and remove irrelevant sections.

ANSWER: As suggested by Reviewer 2, we have removed the irrelevant sections from the discussion of the manuscript.

We noticed you have some minor occurrence of overlapping text with the following previous publication, which needs to be addressed: Cardona, Genís, and Carme Serés. "Grading contact lens complications: the effect of knowledge on grading accuracy." Current eye research 34.12 (2009): 1074-1081. In your revision ensure you cite all your sources (including your own works), and quote or rephrase any duplicated text outside the methods section. Further consideration is dependent on these concerns being addressed.

ANSWER: We have reviewed the text to identify and address all occurrences of overlapping with the previous publication. In addition, we have provided additional references to the text as appropriate.

Please provide additional details regarding participant consent. In the ethics statement in the Methods and online submission information, please ensure that you have specified (1) whether consent was informed and (2) what type you obtained (for instance, written or verbal, and if verbal, how it was documented and witnessed). If the need for consent was waived by the ethics committee, please include this information

ANSWER: We have provided these details in the manuscript and in the online submission information. In the revised manuscript, we have added the following: “Written informed consent was provided by all participants after the nature of the study was explained to them. In this regards, at the start of the study, subjects were only informed that they would be participating in three grading sessions. It was only following the conclusion of the third grading session that the full purpose of the study was explained, including the partial deceit required for the third grading session.”

We note that Figure 1 in your submission contains copyrighted images. All PLOS content is published under the Creative Commons Attribution License (CC BY 4.0), which means that the manuscript, images, and Supporting Information files will be freely available online, and any third party is permitted to access, download, copy, distribute, and use these materials in any way, even commercially, with proper attribution. For more information, see our copyright guidelines: http://journals.plos.org/plosone/s/licenses-and-copyright.

We require you to either (1) present written permission from the copyright holder to publish these figures specifically under the CC BY 4.0 license, or (2) remove the figures from your submission.

ANSWER: We contacted the publisher (Elsevier) and asked permission to publish Figure 1 under the CC BY 4.0 licence. As the book in which the images appear is published on <http://www.sciencedirect.com/>, we were automatically instructed the following:

- Locate the publication containing your desired content on <http://www.sciencedirect.com/science/jrnlallbooks>
- Click on the article/chapter name to access the abstract
- Below the author details, click “Get Rights and Content”
- The Rightslink request page will then be launched (please disable your pop-up blocker)
- Select the way you would like to reuse the content
- Create a Rightslink account if you haven’t done so already
- Accept the terms and conditions and you’re done

Whereupon, the corresponding fee to use the image was determined. After careful consideration, we decided to remove Figure 1 from the submission. We have edited the text accordingly.

**Reviewer #1**

*General Comments*

1) There is the possibility of an order effect, as the labelled images were only presented on the final session. The lack of an appropriate control group which did not have labelled images in the third session make reaching conclusive conclusions regarding the observed change difficult with this as a confound.

ANSWER: The concern of the reviewer is reasonable and adding a control group to the study would have been interesting. Unfortunately, this is no longer possible. However, we believe that, by conducting three grading sessions we partly covered the possible handicap of lacking a control group. Indeed, our findings revealed a moderate to good grading reliability of our participants, when comparing the first and second grading sessions, with no evidence of bias towards higher or lower grades. It was only on the third grading session that a noticeable bias was observed, in which “treated” conditions were consistently awarded lower grades and “untreated” conditions consistently received higher grades than in previous grading sessions. Although we acknowledge that other confounding factors may be at play, we believe it is reasonable to accept the hypothesis that the observed, and consistent, positive or negative bias, originated in the actual “awareness of treatment”. Otherwise, we would have difficulty in interpreting how the order of grading sessions could lead to such consistent findings.

2) The modification of the grading scale from an Ordinal/Discreet scale to one that is a more interval based one using a vertical visual analogue scale. This is different that what the scale would be used for clinically, so discussion on the impact of this modification and thus the output data which was evaluated (on a 100 point scale rather than a 0-4 scale) needs to be discussed

ANSWER: Although this issue was already briefly addressed in the original manuscript, we have elaborated on the discussion of the possible differences between a continuous 100-point scale and a 0-4 discrete scale. The new paragraph reads as follows: “It is interesting to note, however, that this study used a modified version of the Efron scale, presented as a continuous vertical line ranging from 0 mm (healthy eye) to 100 mm (highest possible grade), in contrast to the typical 4 or 5-steps scales commonly employed in clinical practice. Previous researchers have observed that continuous scales are associated with higher grading precision than discrete scales [18,27]. It may be relevant to explore the actual effect of “awareness of treatment” when using a discrete scale in which a 1-step difference may represent a 20 or 25% change in grading.”

3) The use of deception if used typically requires a debrief of subjects after the experiment has concluded, but no details regarding if subjects were debriefed after the study was concluded has been discussed or detailed

ANSWER: The reviewer is correct. We have added the following to the Subjects section of the revised manuscript: “Written informed consent was provided by all participants after the nature of the study was explained to them. In this regards, at the start of the study, subjects were only informed that they would be participating in three grading sessions. It was only following the conclusion of the third grading session that the full purpose of the study was explained, including the partial deceit required for the third grading session.”

*Specific Comments*

Ethics Statement – Incomplete (no ethics number and type of consent has been detailed)

ANSWER: This information is provided in the revised manuscript.

Introduction

- Line 47 – change “optometrist” to “optometrists”

- Line 60 – Remove “Beside” at the beginning of this sentence

ANSWER: Thank you. We have edited the text.

Methods

- How do the authors propose to deal with the confound of the order effect? The changes being seen in the grading may be due to the labelling but may also be due to the learning due to the third test scenario. This could have been alleviated with the use of a control group which did not see these labels, or by randomizing the session where the labels were presented

ANSWER: Please refer to the response provided in the general comments.

- Deception: How were subjects debriefed as to the nature and purpose of the deceit?

ANSWER: Please refer to the response provided in the general comments.

- What is the impact of using the vertical analogue scale in conjunction with the Efron scale? The Efron scale is an ordinal scale, but the vertical analogue scale converts this to an interval scale which defines the differences between the grades to a certain value (a distance on the scale in this instance). As such, the grading is not exactly the same as grading using simply the Efron scale would be, which only has 5 discreet choices which can be made.

ANSWER: Please refer to the response provided in the general comments.

**Reviewer #2**

*Comments are given in the order they occur in the manuscript*

60-62: Citations required.

ANSWER: We have added citations to this sentence.

65: Subjective grading of ocular complications is a complex process involving the comparison of real live images, provided by the slit-lamp….

i. Isn't grading a relatively simple process for the observer? Although the neurological processing involved is undoubtedly complex, it occurs in the background without the observer’s awareness. The whole point of grading systems is that they are simple for clinicians to use, compared to the alternatives.

ii. ....comparison of real-life images, obtained using a slit lamp……

ANSWER: We have replaced “complex” with “relatively simple”. Also, we have edited the sentence as suggested.

67-72: This is a very complex, composite sentence that is difficult to understand. Can it be simplified?

ANSWER: We have edited and simplified this sentence.

79-81: What is the difference between having an advanced knowledge of contact lens complications (high intensity) and having been additionally trained in ocular pathology (specificity)? Seems that these would be the same thing.

ANSWER: This refers to a previous research effort by some of the authors of the present study (Cardona G, Serés C. Grading contact lens complications: the effect of knowledge on grading accuracy. Curr Eye Res. 2009;34:1074-1081). The basis for that study originated in the syllabus implemented in our BSc in Optics and Optometry. In this regards, the course in ocular pathology provided students with knowledge on ocular health and disease, irrespective of whether or not the ocular condition was related to contact lens wear. On the other hand, during the three progressively advanced contact lens courses students were trained to diagnose, grade and manage complications caused by contact lens wear. A student having completed only the ocular pathology course may diagnose a certain ocular complication, but may not be able design an appropriate management strategy if the complication is associated with contact lens wear. Similarly, a student having completed only the contact lens courses will be able to offer a progressively better diagnose, grade and management of the contact lens related complication, but will lack the pathophysiological background to place the condition in the proper context. We believed that the effect of both aspects, although with a moderate amount of overlapping, could be studied independently.

82: ….the Brien Holden……

ANSWER: Thank you. We have edited the text.

92: “The aim of the present study……explore additional sources of bias….”. The Introduction is unsatisfactory because, while it discusses some aspects of using grading scales (intra- and inter- user variability, tendency to use increments etc.), it does little to explain how the outcomes might be subject to bias. Thus, the suggestion here that the aim of this study is “exploring additional sources of bias” is unconvincing. Variability and bias are different phenomena and in the context of this manuscript, the Introduction should be focused on the latter rather than the former.

ANSWER: We have edited the Introduction section of the manuscript extensively to better address the topic of the current research. Thank you for your comment.

92: Is the term "awareness of disease progression" the most appropriate to describe what is being assessed here? What the graders are being told is whether that eye has been treated or not. They do not know anything about how the disease is progressing, just that someone has done something. Consequently the title of the paper may be misleading and should be changed.

ANSWER: The reviewer is correct. We have replaced “awareness of disease progression” with “awareness of treatment”, both in the tittle of the manuscript and elsewhere in the body of the text.

102-109: Please verify that informed consent was obtained from participants.

ANSWER: We have added this information to the Subjects section of the revised manuscript: “Written informed consent was provided by all participants after the nature of the study was explained to them. In this regards, at the start of the study, subjects were only informed that they would be participating in three grading sessions. It was only following the conclusion of the third grading session that the full purpose of the study was explained, including the partial deceit required for the third grading session. “

Did you inform the participants that they should attempt to carry out the grading according to the scale provided and irrespective of the label on the images? This is an important factor as conscious bias may be more pronounced than unconscious bias.

ANSWER: Participants were not specifically directed to the need to grade images without paying attention to the label on the images, that is, the actual relevance of the label within the purpose of this study was not highlighted. Participants were informed that they should grade images as they had done in the previous two sessions, with the only difference that in the third session images corresponded to “treated” or “untreated” conditions. Thus, we believe that bias was unconscious. We have provided more information in the revised manuscript.

In general, the statistical analysis seems overly complicated to achieve the stated aims. Bland & Altman (Bland, J Martin, and Douglas G Altman. 1999. 'Measuring agreement in method comparison studies', Statistical Methods in Medical Research, 8: 135-60) have given clear guidelines on how to investigate these issues and as these methods are being used (though without appropriate citations, please add), it isn’t obvious why additional and more convoluted analyses are necessary. For any given condition (or all conditions combined) all that is needed to test the hypothesis that treatment knowledge affects grading scores is:

i. First, show that the first two repeats (R1 and R2) are unbiased, i.e. the mean difference (R1-R2), for all observers, is not significantly different from zero.

ii. Second, show that the third repeat (R3) is significantly biased, by demonstrating that the mean differences (R1-R3) and/or (R2-R3) are significantly different from zero.

Both i) and ii) above can be achieved by observing the 95% confidence interval for the mean differences and inspection of the scatter of points in the plots may add additional insight. It isn’t clear what extra value is offered by ANOVA, correlation, ICCs etc., so these procedures need to be clearly justified, if they are to be included.

ANSWER: We would like to express our gratitude to the reviewer for this comment. We acknowledge that the statistical analysis was too complicated and redundant. Therefore, we have simplified it by deleting one of the two calculations of grading reliability (see response below), as well as the ANOVA and post-hoc analysis of differences in grading reliability amongst conditions (certainly not relevant for the purpose of the study). We have opted not to remove the intraclass correlation analysis, as this is useful to determine whether participants offered reliable grades, when comparing session 1 and session 2. Non-reliable grades between these two sessions would probably not recommend further analysing the performance of these participants. In addition, we have provided the 95% confidence intervals for the mean differences between grading sessions. Finally, we have added the needed reference to the Bland and Altman paper.

191: Why is the precision quoted for limbal vascularization different from the other features (integer vs one decimal place)?

ANSWER: We have corrected this error. All precision values are quoted with one decimal place in the revised manuscript. The correct value for limbal vascularization is 0.0.

191-194: Can you explain what the difference is between “test-retest differences” and test-retest discrepancy”? These sound like the same thing, but as the SDs being quoted are different, presumably they are not? Is the ”average value of the test-retest differences” not the same as the “average value for the test-retest discrepancy distribution”? Some elaboration and clarification is required.

ANSWER: It is easier to explain it with an example. Average values for test-retest grading differences were 0.2 ± 1.8 (mean ± SD) for bulbar hyperaemia. On the other hand, SD and 95% confidence intervals of the test-retest discrepancy distributions were ±4.2 (±3.7 to ±4.7) for bulbar hyperaemia. As we had 8 images of bulbar hyperaemia and 30 participants, to determine the first set of values (0.2 ± 1.8), we calculated the mean (session 1-session 2) of the 8 images for each participant, and then we calculated the mean and SD of this value including all participants (n=30). This gave us the mean and SD of the differences between session 1 and session 2 for each condition and all participants. Similarly, for the second set of values [±4.2 (±3.7 to ±4.7)], we obtained the mean and 95% confidence intervals of the 30 values of SD obtained from the 8 differences between session 1 and session 2. According to (Efron N, Morgan PB, Jagpal R. The combined influence of knowledge, training and experience when grading contact lens complications. Ophthalmic Physiol Opt. 2003;23:79-85) the widespread of this second set of values gives an indication of grading reliability, with smaller values of SD denoting better reliability.

As reliability was also explored with the intraclass correlation coefficient (ICC), we have opted to delete part of this information: it is a potentially confusing and redundant estimation of grading reliability. In addition, we have edited the text accordingly.

246-267: This section appears to have nothing to do with the study data or outcomes. Please consider deleting it to shorten the text.

268-289: Likewise, this section repeats information already given elsewhere in the manuscript and can be deleted.

ANSWER: We have edited the Discussion of the manuscript to avoid redundancy and to remove irrelevant sections.

344-351: Although this is presented as the Conclusion, most of the text does not rely on or “conclude” from data presented in the manuscript. For example, the text between lines 344 and 348 makes a series of unrelated statements about clinical practice. Please revise to ensure conclusions are relevant and meaningful.

ANSWER: We have edited the Conclusions section of the manuscript as follows: “In conclusion, many sources of bias have been reported to influence grading precision and reliability. The present findings revealed a statistically significant bias, referred to as “awareness of treatment”, in which examiners with moderately reliable grading skills tended to award higher grades to “untreated” conditions and lower grades to “treated” conditions. Further research is required to investigate the clinical significance of this source of bias, particularly given the confined characteristics of the sample and grading conditions of this study.”

The use of the word “novel’ at lines 94 and 348 is unreasonable and should be deleted. Types of bias have always existed; it is only our awareness of them that is new.

ANSWER: We have removed the term “novel” from the manuscript.

Check Ref 11: Spelling of Downie.

ANSWER: Thank you for pointing this typo to us. We have edited the text.
