# Supplementary material for: Awareness of treatment: A source of bias in subjective grading of ocular complications
Source: PLoS One. 2019 Dec 26;14(12):e0226960. doi: 10.1371/journal.pone.0226960 (PMC6932789; doi:10.1371/journal.pone.0226960)
Supplement: Attachment — Submitted filename: Response_to_Reviewers.docx [file pone.0226960.s002.docx]

**Answers to the Reviewers’ comments and list of changes to the manuscript**

Manuscript No.: **PONE-D-19-17494R1**

Title: Awareness of treatment: a source of bias in subjective grading of ocular complications

PLOS ONE

All changes are marked in red in the manuscript. We would like to thank the reviewer for their constructive and positive comments to our manuscript.

**Academic Editor**

1. How were the Intraclass Correlation Coefficients calculated, and are these necessary given repeats one and two were analysed; please justify the use of this analysis.

2. Please report all data with significant decimal places that relates or reflects the precision of the measurement being taken. For example, grading scales to 2 significant places does not reflect the precision of grading scales per se.

3. Please address the issue of the practical and clinical significance of the overall study findings (Reviewer 2).

 ANSWER: Dear Academic Editor, we have addressed these concerns according to the comments and suggestions of the reviewer (please refer to the responses provided below).

**Reviewer #2**: I thank the authors for their responses and have the following further comments.
As this paper is about bias, I wonder whether ICC statistics add any useful information. I agree that establishing repeatability by comparing R1 and R2 is an important step, however aren’t the mean difference between R1 and R2 and the LOAs sufficient to do this? If the authors insist on quoting ICCs, they should specify that the correlations are being calculated between R1 and R2. (lines 173-185). Also presumably ICCs were calculated for each image? Please clarify.

ANSWER: We have edited the Data Analysis section of the manuscript to better describe how ICC values were calculated: “Intraclass correlation coefficients (ICC) (two-way mixed effects, absolute agreement, multiple examiners/measurements model) of session 1 and 2 (test-retest) were calculated to determine grading reliability for each image and the corresponding median and range (minimum-maximum) of values for each ocular condition (comprised of 8 images) was determined”. Even if redundant, we have also added this information to the Results section.

We believe that ICC values are relevant, as they provide an estimation of reliability with a single numerical value that can be compared with published normative data. The Bland-Altman analysis, on the other hand, is very useful in showing the actual distribution of the differences between two measurements, and highlighting any possible bias. We agree with the reviewer that both analyses provide information about reliability, but we believe that this information is not redundant, but complementary. If the reviewer considers that it is indeed unnecessary, however, we would not oppose deleting the ICC analysis from the manuscript.

There is still some inconsistency in precision. LOA values have 2 decimal places as quoted and ICC values have three. Note that the point here is not that they should all be the same, but that they reflect what is reasonable to expect from the characteristics of the measurement. How precise can a subjective grading instrument really be?

ANSWER: The reviewer is correct. We have attempted to be consistent with the number of significant digits throughout the manuscript. Thus, we have followed the recommendations of [Koo TK, Li MY. A guideline of selecting and reporting intraclass correlation coefficients for reliability research. J Chiropr Med. 2016;15:155-163] and [Liljequist D, Elfving B, Roaldsen KS. Intraclass correlation – A discussion and demonstration of basic features. PLoS One. 2019;14:e0219854], as well as of [<https://labwrite.ncsu.edu/res/gh/gh-sigdig.html>], and reported ICC values to two significant digits (such as 0.80 or 0.75) and subjective grading scores to one significant digit, that is, the tenths digit (such as 23.0 or 19.2). We have added the reference [Liljequist D et al] to our manuscript.

I am very confused by the response concerning the distinction between “test-retest differences” and “test-retest discrepancy”. You say “As we had 8 images of bulbar hyperaemia and 30 participants, to determine the first set of values (0.2 ± 1.8), we calculated the mean (session 1-session 2) of the 8 images for each participant, and then we calculated the mean and SD of this value including all participants (n=30). This gave us the mean and SD of the differences between session 1 and session 2 for each condition and all participants. Similarly, for the second set of values [±4.2 (±3.7 to ±4.7)], we obtained the mean and 95% confidence intervals of the 30 values of SD obtained from the 8 differences between session 1 and session 2.” But is it not true that, in general, the grand mean of a data set is invariant to the order in which the columns and rows are averaged? In other words, it should not matter if you average first across the 8 images and then over the 30 participants, or the other way around, the mean (though not the SD) will be the same. Why then, are there two different mean values quoted here?

ANSWER: As suggested by the reviewer and noted in the previous responses to the reviewers “As reliability was also explored with the intraclass correlation coefficient (ICC), we have opted to delete part of this information: it is a potentially confusing and redundant estimation of grading reliability”, this information was considered redundant and deleted from the revised manuscript (R1).

Aiming at clarifying this issue, we agree with the reviewer that the grand mean of a data set is invariant to the order in which columns and rows are averaged. However, please note that the set of values [±4.2 (±3.7 to ±4.7)] did not correspond to the grand mean of grading scores (that is, 8 images per 30 participants) but to the mean (of the 30 participants) of the SD obtained from the differences between R1 and R2 of each condition (set of 8 images) and participant.

In choosing Bland Altman plots to show the data it might help readers see the differences being reported if the same Y-axis scale were used for the treated vs untreated data. Actually, a simple bar chart might be even easier to understand (one bar each for R1, R2 and R3 treated R3 untreated).

ANSWER: Thank you. For clarity, we have added a bar chart figure displaying all results in the same scale (Figure 3).

One final comment is that the quoted difference between R1 and R3 (lines 193-197) all seem to be well within the LOAs for R1 vs R2, irrespective of “treatment” bias. This could reasonably be interpreted to suggest that the typical amount of bias introduce by treatment knowledge is smaller than the normal range of variation found when making successive judgments of the same image. What does this mean for the practical significance of the findings as well as the conclusion that “…more research is needed to investigate the clinical significance.”? Is there not sufficient evidence here to conclude that the effect is unlikely to be of clinical significance?

 ANSWER: The reviewer highlights an important issue. We have further addressed the clinical significance of our findings in the Discussion section of the manuscript (and edited the conclusions of the abstract). The conclusion of the revised manuscript is as follows: “In conclusion, many sources of bias have been reported to influence grading precision and reliability. The present findings revealed a statistically significant bias, referred to as “awareness of treatment”, in which examiners with moderately reliable grading skills tended to award higher grades to “untreated” conditions and lower grades to “treated” conditions. As the study was designed, with very homogeneous characteristics in sample and grading conditions, the clinical significance of this source of bias could not be considered as manifestly superior to the normal range of variation found when making successive judgments of the same image.”
